# Optimal planimetry location for MRI-derived mitral inflow velocity assessment of diastolic function

**DOI:** 10.1186/1532-429X-15-S1-P90

**Published:** 2013-01-30

**Authors:** Shah M Azarisman, Andrew Li, James D Richardson, Dennis T Wong, Seng Keong Chua, Michael Cursaro, Vince Schirripa, Kerry Williams, Ben Koschade, Mitra Shirazi, Julie Bradley, Karen Teo, Matthew Worthley, Stephen G Worthley

**Affiliations:** 1 Cardiovascular Investigation Unit, Royal Adelaide Hospital, Adelaide, SA, Australia

## Background

Diastolic function is almost exclusively assessed using transthoracic echocardiography (TTE), however velocity-encoded phase-contrast imaging permits diastolic evaluation with cardiac magnetic resonance (CMR). However, previous studies have utilized heterogeneous planimetric contour locations to measure mitral valve (MV) inflow velocities and the optimal contour is uncertain. We therefore evaluated CMR MV inflow velocities measured at various regions against TTE to identify the optimal method.

## Methods

Patients with revascularized acute MI and preserved LV systolic function were assessed by 1.5T CMR and TTE at 24-72 hours after presentation. Early and late peak diastolic mitral in-flow velocities were determined at 3 planimetric contour locations: (i) annulus, (ii) MV leaflet orifice, (iii) mid-MV inflow region and the E/A ratio and deceleration times (DT) compared to TTE measurements.

## Results

Twenty-one patients were analyzed and mean LVEF was 56.9±6.9% (TTE) and 58.6±8.3% (CMR). Peak E and A velocities was underestimated by CMR, however E/A showed high correlation with TTE with r2 values of 0.70, 0.69 and 0.74 for the annulus, leaflet and inflow region contours respectively (all p<0.001). Correlation of DT was very strong with r2 values of 0.89, 0.84 for leaflet and inflow region contours respectively (all p<0.001). However the annulus contour poorly correlated with TTE (r2=0.17, p=0.12). Bland-Altman analysis showed the MV leaflet contour to have the best agreement between modalities.

## Conclusions

CMR evaluation of diastolic function is readily achievable and demonstrates high correlation with TTE. However measurements vary according to planimetric contour location, with the greatest correlation noted with MV leaflet whereas the annulus contour demonstrated the weakest relationship.

## Funding

Cardiovascular Investigation Unit, Royal Adelaide Hospital, North Terrace, Adelaide, SA 5000

